# Radiosensitization of calreticulin‐overexpressing human glioma cell line by the polyphenolic acetate 7, 8‐diacetoxy‐4‐methylcoumarin

**DOI:** 10.1002/cnr2.1326

**Published:** 2021-09-02

**Authors:** Amit Verma, Aastha Arora, Anant N Bhatt, Mohan B Arya, Ashok K Prasad, Virinder S Parmar, Bilikere S Dwarakanath

**Affiliations:** ^1^ Institute of Nuclear Medicine and Allied Sciences, Brig. S. K. Majumdar Marg Delhi India; ^2^ SSVPG College Hapur India; ^3^ Bioorganic Laboratory, Department of Chemistry University of Delhi Delhi India; ^4^ Department of Chemistry and Environmental Science Medgar Evers College, The City University of New York Brooklyn New York; ^5^ Central Research Facility Sri Ramachandra Institute of Higher Education and Research Chennai India; ^6^ Present address: PACT & Health LLC, Branford Connecticut, 06405‐2546 USA

**Keywords:** apoptosis, Calreticulin, histone deacetylase inhibitors, ionizing radiation, micronuclei, polyphenolic acetates

## Abstract

**Background:**

Calreticulin (CRT), an endoplasmic reticulum–resident protein generally overexpressed in cancer cells, is associated with radiation resistance. CRT shows higher transacetylase activity, as shown by us earlier, in the presence of the polyphenolic acetates (like 7, 8‐diacetoxy‐4‐methylcoumarin, DAMC) and modifies the activity of a number of proteins, thereby influencing cell signaling.

**Aim:**

To investigate the relationship between CRT expression and radiation response in a human glioma cell line and to evaluate the radiomodifying effects of DAMC.

**Methods and results:**

Studies were carried out in an established human glioma cell line (BMG‐1) and its isogenic clone overexpressing CRT (CROE, CRT‐overexpressing cells) by analyzing clonogenic survival, cell proliferation, micronuclei analysis, and protein levels by Western blotting as parameters of responses. CRT overexpression conferred resistance against radiation‐induced cell death in CROE cells (D_37_ = 7.35 Gy, D_10_ = 12.6 Gy and D_0_ = 7.25 Gy) as compared to BMG‐1 cells (D_37_ = 5.70 Gy, D_10_ = 9.2 Gy and D_0_ = 5.6 Gy). A lower level of radiation‐induced micronuclei formation observed in CROE cells suggested that reduced induction and/or enhanced DNA repair partly contributed to the enhanced radioresistance. Consistent with this suggestion, we noted that CRT‐mediated radioresistance was coupled with enhanced grp78 level and reduced P53 activation–mediated prodeath signaling, while no changes were noted in acetylation of histone H4. DAMC‐enhanced radiation–induced delayed (secondary) apoptosis, which was higher in CROE cells.

**Conclusion:**

CRT overexpression confers resistance against radiation‐induced death of human glioma cells, which can be overcome by the polyphenolic acetate DAMC.

## 1 INTRODUCTION

Glioma is the most common type of human cancer that is refractory to a variety of therapies with a 5‐year survival rate of less than 10% post treatment.^1^, ^2^ Various forms of radiotherapy with temozolamide (an alkylating agent and DNA methyl transferase inhibitor) is the current standard of care in the management of malignant gliomas following surgery that modestly enhances patient survival.^3^ Despite significant technological advances, success of radiation therapy is limited due to the radioresistance of glioma cells linked to alterations in multiple signaling pathways related to DNA damage and repair, apoptosis, cancer stem cells proliferation, and self‐renewal.^4^ Consequently, radiosensitizers targeting these pathways have shown promising clinical outcome, enhancing the specificity and efficacy.^5^ However, acquired drug resistance and undue normal tissue cytotoxicity pose limitations to the widespread use of these agents preventing clinical benefits.^6^ Therefore, there has been a considerable amount of effort in identifying and/or designing novel drugs or approaches which are effective in the management of radiation therapy.

**FIGURE 1:**
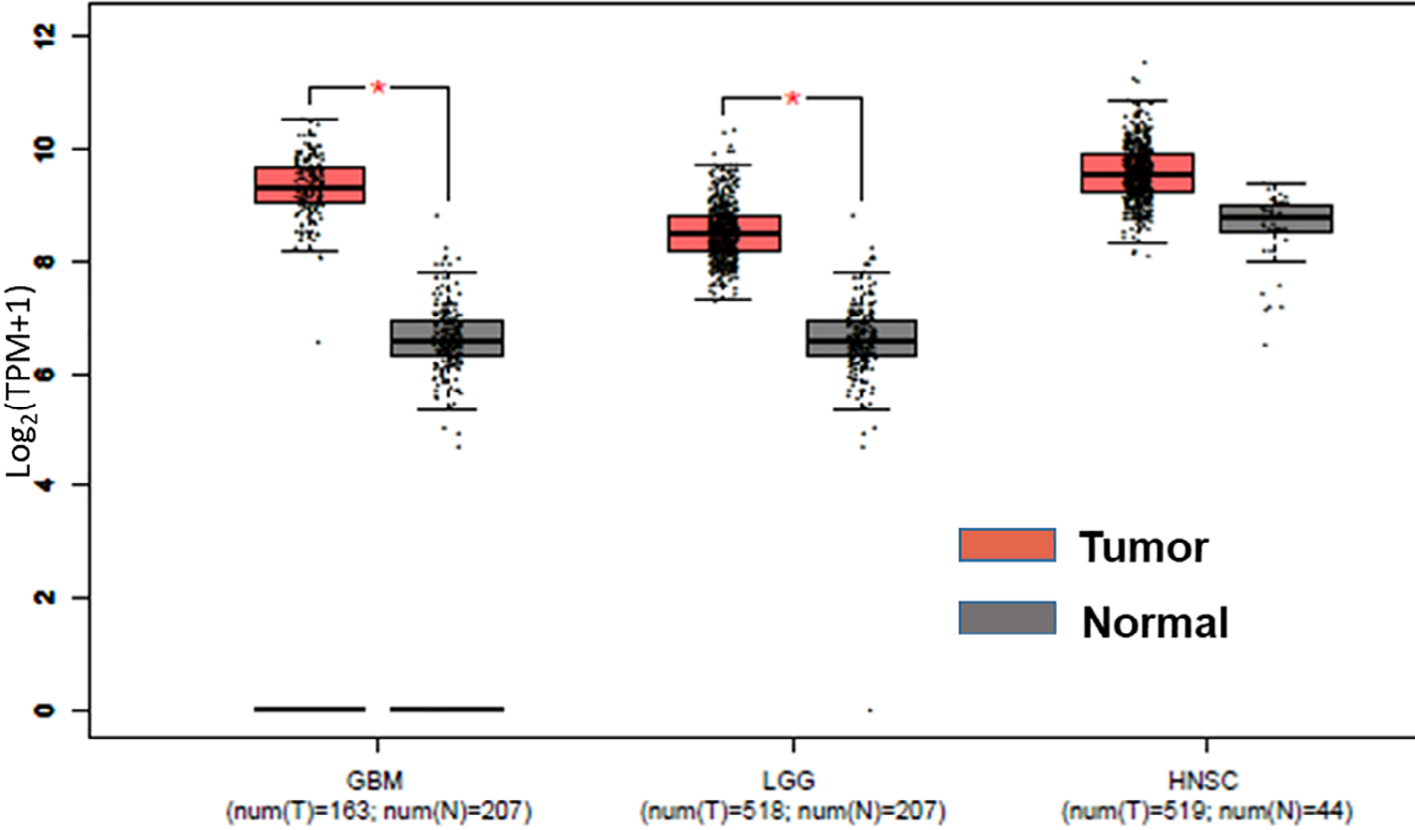
Human glioma and head and neck cancers express a higher level of calreticulin gene compared to normal tissues. Expression of calreticulin gene in human glioma and head and neck cancers analyzed using the online tool—Gene Expression Profiling Interactive Analysis (GEPIA) (http://gepia.cancer‐pku.cn/). Each dot represents expression of samples. Y‐axis represents log2 (TPM + 1) as the expression level, where TPM is transcripts per million; *p* < .01

Calreticulin (CRT), a calcium‐binding, endoplasmic reticulum–resident protein, has emerged as an important molecule of interest due to its multifunctional role in cells including maintenance of intracellular calcium homeostasis, molecular chaperoning function, cell adhesion, migration, apoptosis, etc.^7^, ^8^ Studies with CRT‐knockout mice have revealed its indispensable role in tissue growth and development.^9^ Role of CRT has been implicated in a variety of disease conditions like heart disease, immune dysfunction, wound healing, tissue repair, cancer, etc.^10^ A strong correlation exists between CRT expression and tumorigenesis progression, and metastasis with overexpression in many types of solid tumors.^11^, ^12^ Gene expression profiling interactive analysis (GEPIA) shows high CRT expression in gliomas (low grade as well as glioblastoma multiform, GBM) and head and neck carcinomas (Figure 1). Recent studies have shown that CRT expression is a powerful prognostic biomarker reflecting enhanced antitumor immune response in patients with non–small‐cell lung carcinoma (NSCLC) and acute myeloid leukemia.^13^, ^14^ In response to stress like ionizing radiation (IR), CRT translocates to the surface of dying cancer cells and acts as “eat‐me” signal for antigen‐presenting cells (APCs). The APCs phagocytose the dying cells that leads to antigen presentation and tumor‐specific cytotoxic T lymphocytes response.^15^ CRT has been shown to regulate P53‐mediated UV‐induced apoptosis by affecting the rate of P53 degradation and nuclear localization in mouse embryonic fibroblast cells.^16^, ^17^Although few studies have highlighted the role of CRT in determining the cellular responses to IR, further studies demonstrating a relationship between CRT expression and IR‐induced glioma cell death are merited.

Protein acetylation regulates a variety of cell functions including chromatin remodeling and gene expression through transactivation, protein–protein interactions, and stabilization of the target proteins.^18^, ^19^, ^20^ Canonical protein acetylation system involves lysine acetyltransferases (KATs) and deacetylases (KDACs) and acetylating/deacetylating lysine residues on histones and nonhistone proteins. KDACs are upregulated in many cancers such as leukemia, glioblastoma, melanoma, carcinoma, etc.^21^, ^22^ Consequently, KDAC inhibitors that induce apoptosis, cellular differentiation, and cell cycle arrest of cancer cells have emerged as promising anticancer therapeutics that target the altered epigenetic regulation in tumors.^23^ However, acute side effects associated with HDACi like gastrointestinal toxicity, cardiac arrhythmia, thrombocytopenia, anemia, fatigue, nausea, vomiting, etc. limit their use as radiosensitizers in clinics.^24^ Therefore, novel drugs or approaches which simulate effects similar to KDAC inhibitors, but are associated with lesser toxicity, are required to improve the efficacy of radiotherapy.

Our earlier investigations have established the transacetylase function of CRT wherein it efficiently transfers acetyl group from polyphenolic acetates (PAs) to target proteins while acetyl co‐enzyme was a weak donor.^25^, ^26^, ^27^ In vitro studies have established that among a variety of acetylated polyphenols with a varying number of acetyl group side chains, 7, 8‐diacetoxy‐4‐methyl coumarin (DAMC) is an efficient acetyl group donor, acetylating the target proteins (enzymes) like cyto‐P‐450, NADPH‐cyto c reductase, glutathione S transferase, and nitric oxide synthase modulating their activities and associated physiological effects.^28^ Acetoxy drug: CRT transacetylase (CRTase) protein acetylation system extends the realm of protein acetylation beyond the KAT/KDAC system. Our earlier studies have shown that DAMC sensitizes the human glioma cell line (BMG‐1) by impairing the removal of radiation‐induced DNA double‐stranded breaks thereby enhancing mitotic and interphase death.^29^ The radiosensitization correlated with hyperacetylation of histone H3 lysine (9/14) by DAMC is similar to radiosensitizing effects of classical HDAC inhibitors.^30^ Although these circumstantial evidences suggest a role for CRT:DAMC in determining cellular responses to IR, a causal relationship between CRT:DAMC‐mediated protein acetylation and radiosensitivity has not been established. Therefore, the present studies were undertaken in human glioma cell line (BMG‐1) and its isogenic cells overexpressing CRT (CROE), to further establish the role of CRT in the radiosensitizing effects of DAMC. Our results show that CRT expression leads to the radioresistance of BMG‐1 cells which are coupled with enhanced grp78 level and reduced P53 activation–mediated prodeath signaling. Further, radiosensitization by DAMC was significantly higher in the CRT‐overexpressing CROE cells, suggesting that DAMC could be a potential adjuvant in enhancing the efficacy of radiotherapy in radioresistant tumors overexpressing CRT.

## 2 MATERIAL AND METHODS

### 2.1 Material

Dulbecco's Minimum Essential Medium (DMEM), phosphate buffered saline (PBS), Penicillin G 50000 unit/l, streptomycin 38 850 unit/l and Nystatin 9078 unit/l, dimethylsulfoxide (DMSO), ethylene‐di‐amine‐tetra acetate (EDTA), HEPES, Hochest‐33 258 (H33258), propidium iodide (PI), RNAase, fetal bovine serum, and Fura‐2 am were procured from Sigma chemicals Co. (St Louis, USA). 6‐NBDG was purchased from Thermofisher, USA. Primary antibody anti‐CRT (recognizing C‐termini of CRT), anti‐grp78, acetylated histone H3 antibody (lysine9/ 14), secondary antibody (goat antirabbit, HRP conjugate), secondary antibody (IgG1 whole molecule with HRP conjugate), and secondary antibody (goat antirabbit, FITC conjugate) were procured from Santacruz Biotechnology Inc, CA, USA. Antiacetylated P53 (lysine 382) antibody was purchased from Cell Signaling, Danvers, MA, USA. Antifade Reagent was purchased from Molecular Probe. 7, 8‐Diacetoxy‐4‐methyl‐coumarin (DAMC) was synthesized at the Department of Chemistry, University of Delhi. Gamma ray irradiation (source: ^60^Cobalt) was carried by using Bhabhatron‐II Teletherapy machine (Panacea, Medical Technologies Pvt. Ltd., Bangalore, India). GraphPad Prism version 7.00, GraphPad Software, La Jolla, CA, USA.

### 2.2 Source of human tumor cell lines

Human cerebral glioma cell line (BMG‐1; diploid, wild‐type P53) was established in the Department of Biophysics, National Institute of Mental Health and Neuro Sciences, Bangalore, India, and was used in the study.^31^ Cells were periodically checked for ploidy, growth rate, and clonogenic potential (plating efficiency) and revived after 40 passages. Calreticulin‐overexpressing BMG‐1 cells (CROE) were prepared as described earlier.^32^ The cells were maintained as monolayers at 37°C in 25 cm^2^ tissue culture flasks (Tarsons, India). The cells were maintained in Dulbecco's modified Eagle medium (DMEM) supplemented with 5% fetal bovine serum, HEPES, sodium bicarbonate, and antibiotics. Cells were passaged routinely in exponential growth phase twice a week using 0.05% trypsin solution (with 0.02% EDTA, 5.5 mM glucose, and 0.002% phenol red) in phosphate‐buffered saline (PBS). All experiments were carried out in exponentially growing cells.

### 2.3 Drug treatment

DAMC was dissolved in DMSO and diluted in Hanks Basal salt solution (HBSS) before filter sterilizing and added to the cells in the required concentration after serial dilution in growth media for 24 h.

### 2.4 Irradiation

A ^60^Cobalt source was used (at 80 cm source to surface distance and 35 × 35 cm field with a dose rate of 2 Gy/min) in the study. The cells were pretreated with DAMC (100 μM) for 22 h followed by exposure to ionizing radiation and kept for additional 2 h in the presence of the drug.

### 2.5 Macrocolony assay

Cells ranging from 150‐4000 [cells/cm^2^] (depending on the treatment) were plated 24 h before treating with varying concentrations of DAMC in DMEM at 37°C and irradiated according to the protocol discussed above. After the treatment, cells were replaced with fresh growth media and incubated in a CO_2_ incubator for 7‐8 days. Colonies (more than 50 cells) were fixed with methanol and stained with 1% crystal violet and were counted to calculate the plating efficiency (PE) and the surviving fraction (SF).

$$\mathrm{PE}=\left(\mathrm{No}.\text{of colonies counted}/\mathrm{No}\;\text{of cells plated}\right)\;x\;100$$

SF=PET/PEC,

where PET is the plating efficiency of the treated group and PEC is the value of the control.

Survival curves were generated by applying linear quadratic regression model using GraphPad Prism Software. Survival fraction (SF) was obtained by interpolating the linear quadratic survival curves. α/β ratio was calculated by the following equation:

$$\alpha /\beta =\exp\left[-\left(\mathrm{\alpha D}+\text{βD2}\right)\right],$$

where D is a radiation dose (Gy). From this regression curves, D_37_, D_10_, and D_0_ values were obtained by GraphPad Prism software. Using D_10_ (dose inducing 90% cell death) values, sensitization enhancement ratio (SER), indicating DAMC‐induced enhancement in radiosensitivity, was calculated by the following equation:

$$\mathrm{SER}\;\mathrm{at}\;10\;\mathrm{Gy}\;\left(\mathrm{SER}=D_{10}\left[\text{Radiation}\right]/D_{10}\left[\text{DAMC}+\text{Radiation}\right]\right)$$

### 2.6 Cell proliferation

Following different treatments as described above, cells were harvested at every 24 h time interval. Both floating and attached cells were counted and fixed in 70% chilled ethanol for the analysis of cell cycle distribution. Cell proliferation was calculated by computing the increase in cell number, and an index of proliferation P was calculated as:

$$P=N_{t}/N_{0}.$$

where, N_t_ = number of cells at any postirradiation time “t.”

N_0_ = number of cells at the time of treatment.

### 2.7 Cell cycle distribution

Flow cytometric measurements were performed with 70% ethanol fixed cells at 4°C for at least overnight and stained with intercalating DNA fluorochrome propidium iodide (PI). Fixed cells were washed with PBS, treated with 200 μg/mL RNAse‐A for 30 min at 37°C, and stained with 50 μg/ml propidium iodide (Sigma, USA) in PBS. Data were acquired on a flow cytometer (FACS‐Calibur, Becton‐Dickinson, CA, USA) using the CellQuestPro software (version 3.0.1; Becton Dickinson, CA, USA) and, ModFit LT (version 2.0; Verity Software House, Inc., USA) software was used for the cell cycle analysis.

### 2.8 Micronuclei expression

Cells were washed twice in phosphate buffered saline (PBS, pH 7.2) and fixed in Carnoy's fixative (methanol:acetic acid; 3:1) at 4°C for 2‐4 h. The fixed cells were spread on clean prechilled microscopic slides. Following overnight air drying, slides were stained with 10 μg/mL Hoechst‐33 258 in phosphate buffer [Na_2_HPO_4_.2H_2_O, Tween‐20 0.5%, and 0.1 M citric acid] in the ratio 9:1, final pH 7.4 for 30 min in dark at room temperature as described earlier.^25^ After washing the excess stain with distilled water followed by PBS, the slides were mounted in PBS‐glycerol (1:1) and observed under fluorescence microscope (Olympus BX60, Japan) using UV excitation filter. A total of 1000 cells were scored per group.

The frequency of cells with micronuclei, called the M‐fraction (MF), was calculated as:

$$\mathrm{MF}\;\left(\%\right)=N_{m}/N_{t}\;x\;100$$

where N_m_ is the number of cells with micronuclei and N_t_ is the total number of cells analyzed.

Since proliferation influences radiation‐induced micronuclei expression as they are expressed in postirradiation daughter cells and radiation causes perturbations in cell cycle progression, we normalized the values of cells with micronuclei linked to proliferation as follows:

$$\text{Normalized percent micronuclei}=\text{Percent micronuclei}\;\left(\mathrm{MF}\%\right)/\text{Proliferation}\;\left(P\right),\text{where}\;P=N_{t}/N_{o}.$$

### 2.9 Apoptosis

Flow cytometric measurement of cellular DNA was performed with ethanol fixed cells as described earlier.^32^ Presence of sub G_1_ (hypodiploid) population is indicative of apoptotic cell death and was calculated using Modfit software (Becton and Dickinson), which was subsequently reconfirmed from regional statistics in bivariate plots of DNA versus forward scatter or side scatter.

### 2.10 Intracellular calcium (Ca^**2**+^) levels

The intracellular levels of calcium in CROE cells and parental BMG‐1 cells were measured by fura‐2 that binds to the cytosolic calcium. The cells were grown on cover slips and, 48 h later, were washed with PBS and stained with Fura‐2 (3 μM) for 15 min. The samples were washed, and antifade mounting solution was applied to prevent bleaching. Cells were visualized using fluorescence microscope with UV excitation (Zeiss, Germany).

### 2.11 6‐NBDG staining

The BMG‐1 and CROE cells were incubated with 6‐NBDG (50 μM, 6‐(N‐(7‐Nitrobenz‐2‐oxa‐1,3‐diazol‐4‐yl) amino)‐6‐deoxyglucose) prepared in phosphate buffered saline (PBS) for 30 min. Cells were then harvested by trypsinization and washed twice with cold PBS and resuspended in PBS. Data were acquired on a flow cytometer (LSR II, Becton Dickenson, CA, USA) using a blue LASER (488 nm) excitation, and the green fluorescence (535 nm) was recorded. Analysis was carried out using FACS Diva software (Becton Dickenson, CA, USA).

### 2.12 Immunocytochemistry

The CRT was measured by fluorescence microscopy using anti‐CRT antibody. The cells were plated with density of 0.2 × 10^6^ on glass cover slips and incubated overnight at 37° C in 5% CO_2_. Next day, the cells were fixed and permeabilized in acetone:methanol (1:1) at ‐20° C for 15 min. After fixation and permeabilization, the cells were washed with chilled TTBS (20 mM Tris, 150 mM NaCl, 0.2% Tween 20; pH 7.4) and blocked with 5% goat serum in TBST for 1 h at room temperature. The cells were then incubated with anti‐CRT antibody in dilution buffer (1% Bovine serum albumin in TTBS) for 1 h at room temperature followed by washing. FITC‐labeled secondary antibody (1:1200) was then added, and the cells were incubated for 1 h at room temperature. The cells were washed and mounted with an antifade reagent containing DAPI. The cells were examined under the fluorescence microscope, and images were captured using 40× objective.

### 2.13 Western blotting

Preparation of cell lysates and immunoblot analysis from untreated and IR (2 Gy)‐treated cells were harvested after 48 h and lyzed in ice cold RIPA lysis buffer (Tris‐HCl: 50 mM, pH 7.4, NP‐40:1%, Na‐deoxycholate: 0.25%, NaCl: 150 mM, EDTA: 1 mM, PMSF: 1 mM, aprotinin 1 μg/mL, leupeptin 1 μg/mL and pepstatin: 1 μg/ml, Na_3_VO_4_: 1 mM, NaF: 1 mM). The protein content in the lysates was measured by BCA protein assay. Protein (50‐60 μg) was resolved on 15% SDS‐PAGE and electroblotted onto a PVDF membrane (Amersham). The membrane was then incubated in 4% skimmed milk for 1 h followed by primary antibody incubation anti‐CRT, anti‐grp78, acetylated histone H3 antibody (lysine9/ 14), and antiacetylated P53 (lysine 382) for 2 h. After washing, corresponding secondary antibody (horseradish peroxidase conjugate) was added and incubated for 1 h. After washing, the blots were developed using ECL chemiluminescence detection reagent (Pierce). Membranes were stripped in stripping buffer (25 mM Glycine, 1% SDS, pH 2 adjusted with HCl) for 1 h, washed twice in TTBS for 10 min eachm and reprobed with β‐actin (cytoplasm) as loading control.

### 2.14 In vivo tumor transplantation

The inbred nude male mice were used in this study and were obtained from the Institutes' central animal facility and weighed 20‐25 g at the time of tumor implantation. The BMG‐1 and CROE cells were implanted by subcutaneous injection of 10 × 10^6^ cells (in 0.1‐0.15 mL volume) into the right hind leg. Tumor volume was calculated using the formula: V = π/6 (d1 × d2 × d3), where d1, d2, and d3 are the three orthogonal diameters measured with the help of a Vernier calipers. Experiments were performed when the tumors had attained a volume of 100 mm^3^ (10 days after implantation). Animals were sacrificed using cervical dislocation method, when the tumor reached a volume to avoid tumor burden–related discomfort to the animal as per the UKCCCR guidelines for the welfare of animals in experimental neoplasia.

### 2.15 Statistical analysis

All the experiments were carried out in triplicates. Data are expressed as mean values with SD. The statistical significance between groups was calculated using two‐tailed Student's “t”‐test.

## 3 RESULTS

### 3.1 CRT‐overexpressing (CROE) glioma cells show altered calcium status, stress response, glycolysis, and accelerated tumor growth

**FIGURE 2:**
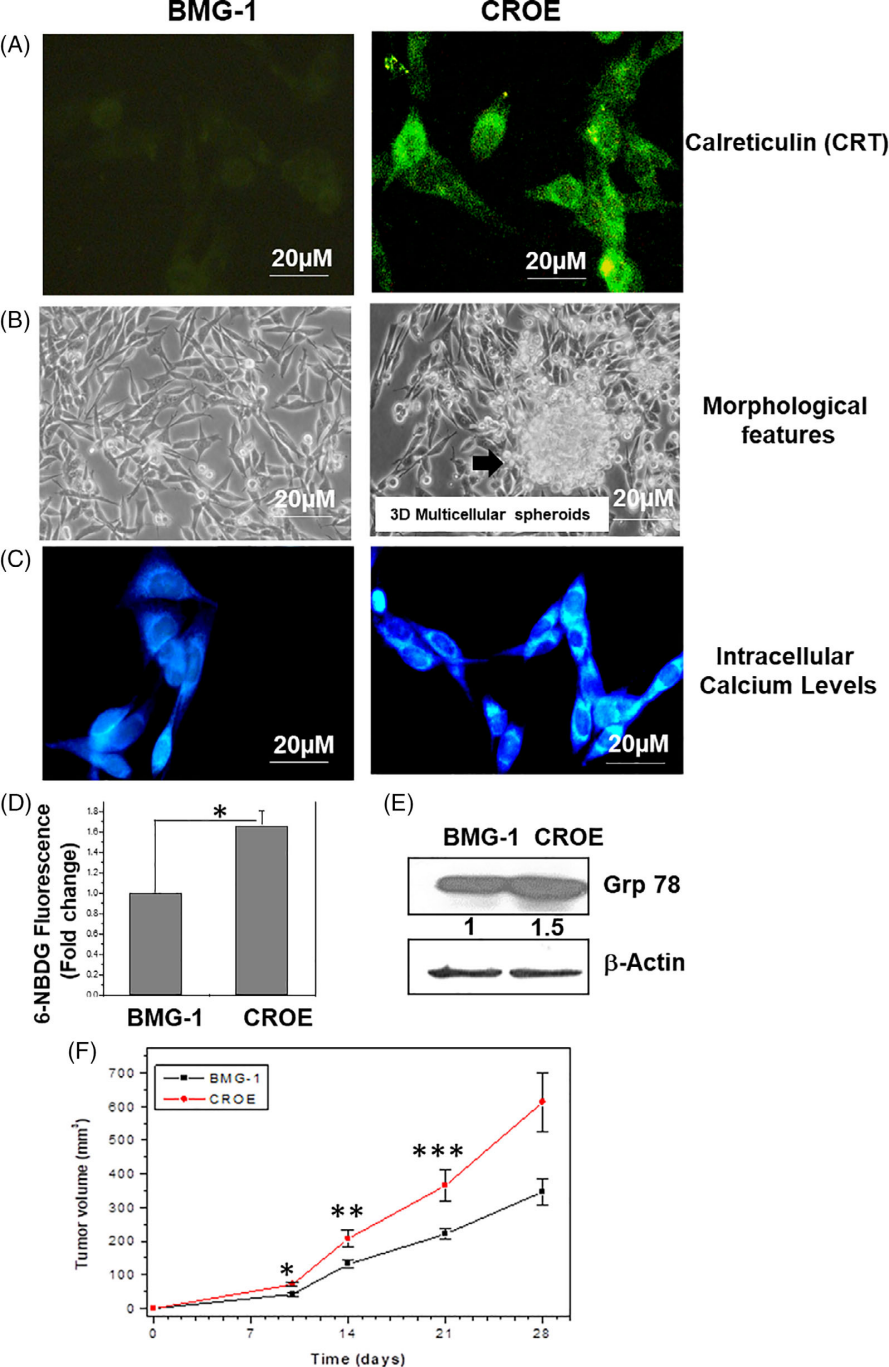
CRT overexpression altered certain phenotypic behavior of cells. Characteristics of wild type (BMG‐1) and isogenic calreticulin (CRT) overexpressing (CROE) human glioma cells. (A) Immunofluorescence of CRT protein in BMG‐1 and CROE cells. The CRT protein was probed using antibody recognizing C‐termini of CRT protein, labeled green. (B) Morphological features of BMG‐1 and CROE monolayer cultures showing tendency for spheroid formation in CROE. (C) Distribution of intracellular calcium (Ca^2+^) in BMG‐1 and CROE cells labeled by Fura2‐AM, labeled blue. (D) Measurement of glucose uptake in BMG‐1 and CROE cells using fluorescent glucose analogue 6‐NBDG. The data shown are the mean value of three independent experiments. *p < .05. (E) Immunoblots analysis of grp78 protein level in BMG‐1 and CROE cells; β‐actin was used as loading control. (F) Growth of BMG‐1 and CROE tumors grafted in nude mice. Mice were injected with 10 × 10^6^ cells; n = 3 for BMG‐1 cells and n = 4 for CROE cells. *p < .001, **p < .005, ***p ≤ .05. Time to reach 300 mm^3^ volume was 25.5 days in BMG‐1 and 18 days in CROE

High CRT level is known to influence the expression of many structural and functional proteins that alter the morphological features and cellular responses to stress.^11^ CRT overexpression in CROE cells was confirmed by immunoblotting and immunocytochemistry using anti‐CRT antibody–recognizing C‐termini of CRT protein (Figure 2A).^32^ CRT expression was mainly localized in the cytoplasm. Although the gross morphology of CROE cells was not different from the parental BMG‐1 cells, they tended to form few spheroid‐like structures, which were not observed in BMG‐1 cells (Figure 2B). CRT, besides facilitation of CROE cells, showed a higher level of intracellular Ca^2+^ and grp 78, an endoplasmic reticulum chaperone (Figure 2C,E) in line with the well‐known regulation of intracellular Ca^2+^ homeostasis and endoplasmic reticulum Ca^2+^ storage capacity, besides chaperoning of misfolded proteins.^7^

Interestingly, an increase in the glucose uptake was also noted in CROE cells as compared to the parental BMG‐1 cells (Figure 2D). CROE cells grafted in nude mice grew rapidly as compared to the parental BMG‐1 cells with time to reach 300  mm^3^ volume during 26 days in BMG‐1 and ~18 days in CROE (Figure 2F) suggesting an aggressive nature of CROE cells. These results indicate that CRT overexpression (CROE) partly altered the biological behavior of parental BMG‐1 cells and may influence their response to stress such as ionizing radiation (IR).

### 3.2 Overexpression of CRT induces resistance against radiation‐induced cell death

To study the effects of CRT on cellular response to radiation, we investigated various cytotoxic parameters and the potential contributing factors.

**FIGURE 3:**
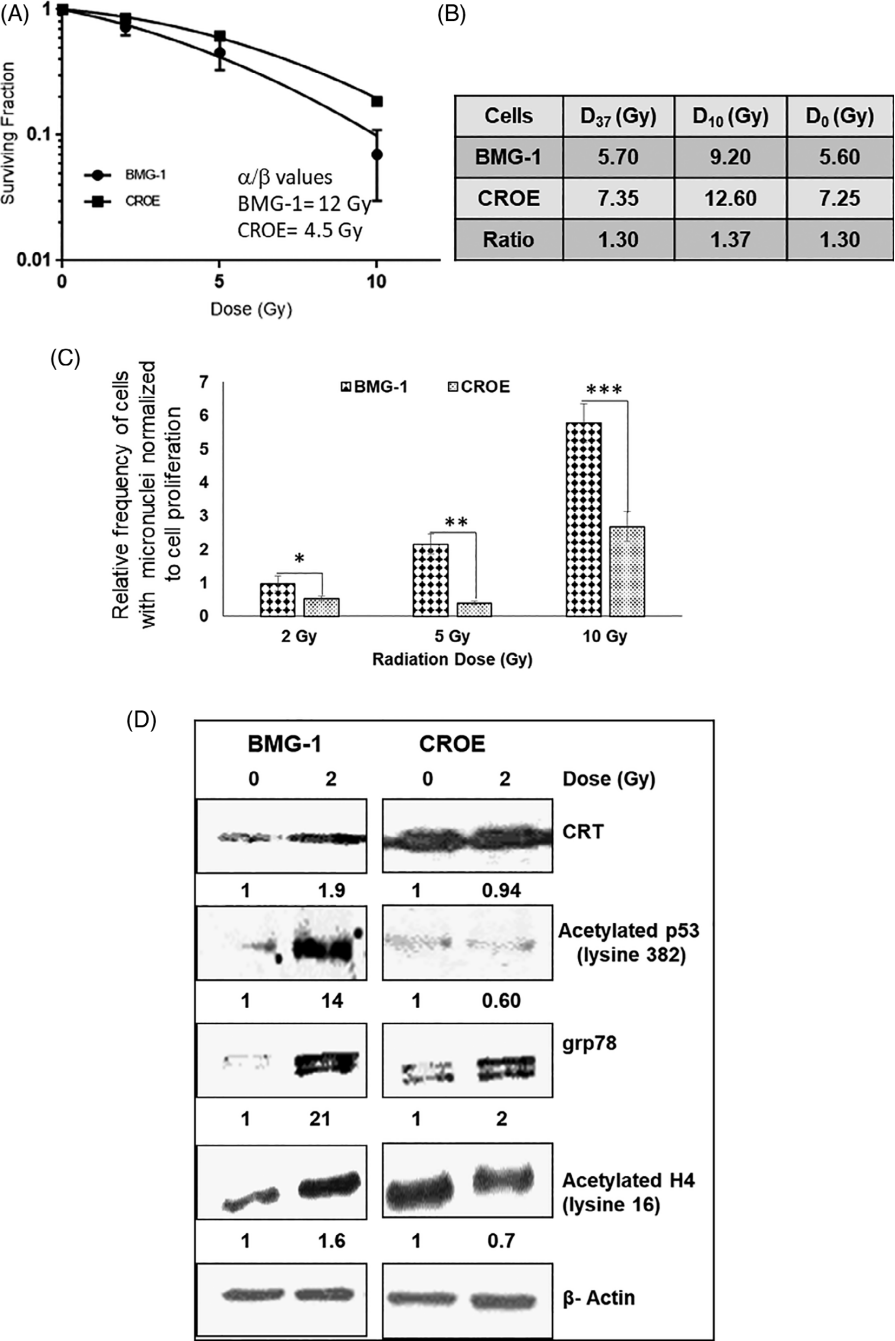
CRT overexpression conferred resistance. (A) Dose‐dependent changes in clonogenicity of BMG‐1 and CROE cells following ^60^Co gamma radiation. (B) Values of radiation response parameters viz. D37, D10, and D0 derived from Figure 3A and the ratios (Dx CROE/ Dx BMG‐1; where x is D37, D10, or D0 values) indicate the fold increase in radioresistance of CROE cells compared to BMG‐1 cells. (C) Radiation‐induced cytogenetic damage (micronuclei frequency) observed at 48 h post irradiation normalized to cell proliferation (please see Methods for details). The data shown are mean values of three independent experiments. The significance was calculated between irradiated BMG‐1 and CROE cells. *p < .05, **p < .002, ***p < .001. (D) Changes in acetylated p53 (lysine‐382) and histone H4 (lysine 16) analyzed at 48 h post irradiation

*Clonogenic survival*: The dose response of clonogenic survival showed that CROE cells were relatively more resistant than the parental BMG‐1 cells (Figure 3). The α/β values (indicative of radiation response in the linear quadratic model) were 12 and 4.5, respectively, for BMG‐1 and CROE cells (Figure 3A). Other radiobiological parameters viz. D_37_ (dose at 37% survival), D_10_ (dose at 10% survival), and D_0_ (the slope of the exponential portion of the dose–response curve) were, respectively, 7.35 Gy, 12.6 Gy, and 7.25 Gy for CROE cells as compared to 5.70 Gy, 9.2 Gy, and 5.6 Gy for BMG‐1 cells (Figure 3B). These results suggest that CRT overexpression provides survival advantage to CROE cells indicating its potential role in rescuing cells from radiation‐induced cell death. These observations are at variance with the results reported earlier in U251 glioma cells where CRT overexpression resulted in sensitization.^33^

*Micronuclei expression*: Mitotic death linked to cytogenetic damage is an important mode of radiation‐induced cell death. Therefore, we investigated radiation‐induced micronuclei formation (a form of cytogenetic damage observed in postmitotic cells). Since micronuclei expression is linked to proliferation as micronuclei are expressed only in the postirradiation daughter cells, we normalized the micronuclei frequency to cell proliferation. The normalized values of micronuclei induction at 48 h post irradiation were significantly lower in CROE cells at all three radiation doses as compared to the parental BMG‐1 cells (Figure 3C), thereby suggesting that decrease in the mitotic death is a significant contributing factor for the enhanced radioresistance in CROE cells.

### 3.3 Protein levels

To investigate the potential contributing factors for CRT‐mediated radioresistance, we analyzed the level of few proteins implicated in the prodeath and prosurvival using immunoblotting. Radiation‐induced increase in the level of acetylated P53 implicated in damage‐induced cell death regulated by P53 was nearly 14‐fold lower in CROE cells as compared to BMG‐1 cells 48 h post irradiation (Figure 3D).^34^ We also observed increased grp78 (a molecular chaperone) levels in CROE cells compared to BMG‐1, suggesting a higher level of unfolded protein response implicated in rescuing cells from radiation‐induced cell death.^35^ The level of acetylated H4 (lysine 16) was ~2‐fold higher in BMG‐1 cells, while no significant change was noted in CROE cells. However, the endogenous level of CROE cells was higher than that of BMG‐1 cells.

### 3.4 Radiosensitization by DAMC is higher in CRT‐overexpressing CROE cells

**FIGURE 4:**
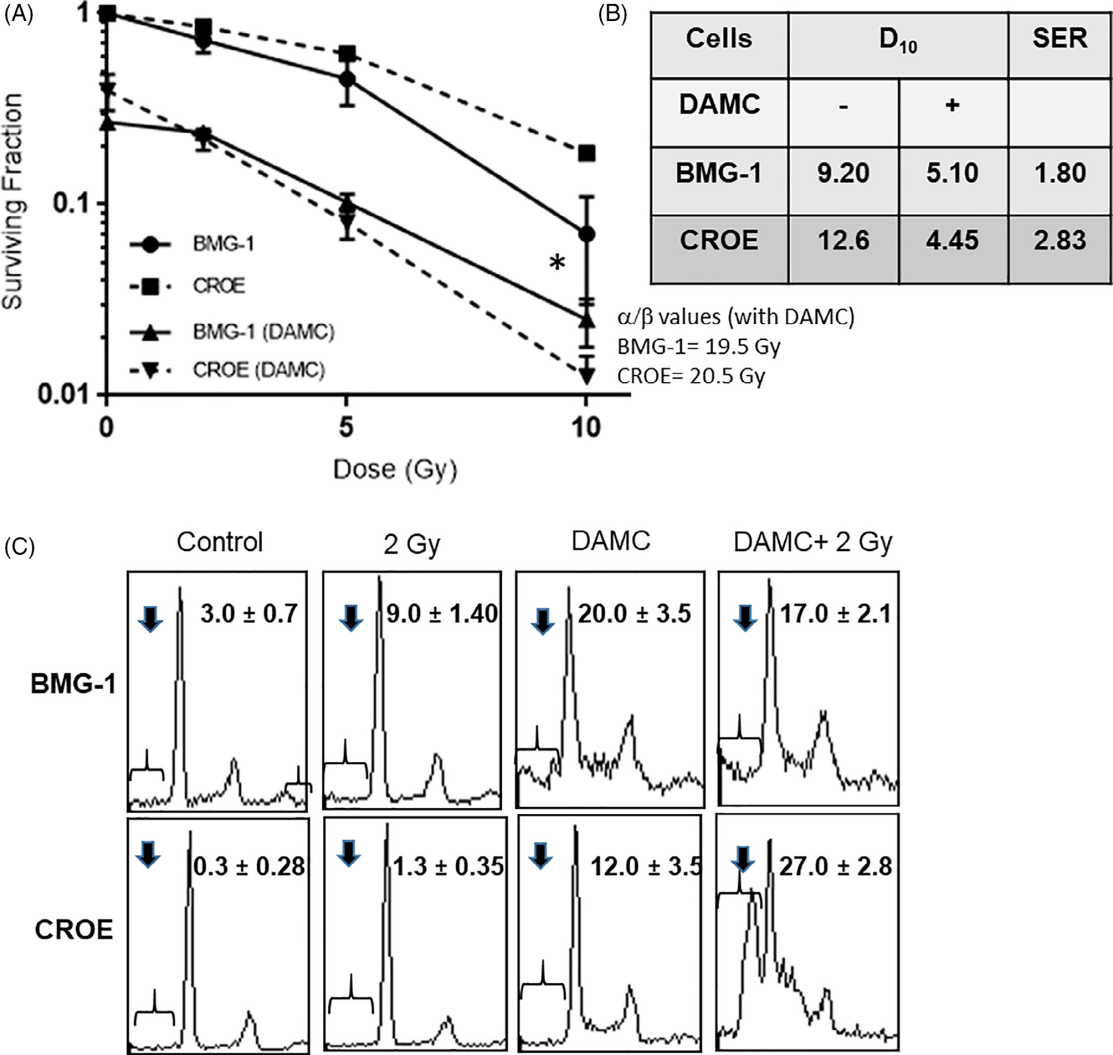
Radiosensitization by DAMC was higher in CRT‐overexpressing CROE cells. (A) Dose response of clonogenic survival in BMG‐1 and CROE cells. (B) D10 (dose inducing 90% cell death) values and sensitization enhancement ratio SER at 10 Gy (SER = D10[Radiation]/D10[DAMC+ Radiation]) indicating DAMC‐induced enhancement in radiosensitivity. (C) Flow cytometric DNA histograms showing hypodiploid population (suggestive of apoptosis) observed at 48 h post irradiation. The values in the inset are mean (±1 SD) of three independent experiments

Our earlier studies have shown that DAMC has both cytotoxic and radiosensitizing effects in BMG‐1 cells and the radiosensitizing effects are additive in nature.^29^ The radiosensitization by DAMC was profoundly higher in CROE cells as compared to BMG‐1 cells (Figure 4A), and the shoulder on the dose–response curve was abolished in CROE cells (Figure 4A). Although the α/β values for BMG‐1 and CROE cells treated with DAMC were nearly similar (19.5 and 20.5), this meant an increase of 55% in the value from cells without DAMC treatment in case of BMG‐1 cells (from 12.5 to 19.5) and nearly 480% in CROE cells (from 4.5 to 20.5). Accordingly, the radiosensitization was supra‐additive in nature with a sensitization enhancement ratio (SER) value of 1.80 at 10% survival in BMG‐1 cells, while it was 2.9 in CROE cells (Figure 4B).

*Apoptosis*: Interphase death (mainly in the form of apoptosis) can also contribute to the loss of clonogenicity following irradiation. Using flow cytometric DNA measurements, we analyzed the hypodiploid population (suggestive of apoptotic cells) at 48 h after irradiation, as ionizing radiation–induced apoptosis is a secondary event at low and moderate doses of low LET ionizing radiation particularly in epithelial tumor cells.^36^ DAMC and radiation had a supra‐additive effect on the induction of hypodiploid cells in CROE cells, while less than additive effect was noted in BMG‐1 cells (Figure 4C). Taken together, these results suggest that DAMC enhances radiation‐induced interphase (apoptosis) as well as mitotic death in these cells.

## 4 DISCUSSION

Although a higher level of calreticulin (CRT) expression is observed in a wide variety of human tumors including low‐ and high‐grade gliomas, a strong correlation between CRT level and clinical outcome (overall survival) has not been observed. This could arise on account of the contributions from other biological behavior of tumors, besides CRT levels that contribute to the growth and response to therapy, which could vary among different tumors and among patients harboring the same tumor. Therefore, in the present studies, we investigated the influence of CRT on physiological and radiobiological responses, using isogenic cell lines of a human glioma (BMG‐1) expressing low and high levels of CRT. Results suggest that CRT could confer resistance against radiation‐induced cell death, similar to the prevention of oxidative stress and cell death in renal epithelial cells reported earlier.^37^ Enhanced glycolysis (glucose uptake; Figure 2) and GRP 78 level known to confer resistance against stress including radiation may contribute to the CRT‐linked radioresistance.^38^, ^39^ However, our observation is at variance with the results reported earlier in U251 glioma cells where CRT overexpression resulted in radiosensitization, suggesting that the effect of CRT on radiation response could be cell‐type dependent and needs further investigations.^33^ Interestingly, the polyphenolic acetate DAMC that has been shown as a preferred acetyl group donor for the CRT‐mediated protein acetylation sensitized the CRT‐overexpressing cells (CROE) to a higher degree than the parental cells, suggesting its potential as an adjuvant to radiotherapy, particularly in CRT‐linked radioresistant tumors.^27^

Present study demonstrates that CRT overexpression changes intracellular physiology and enhances tumorigenic potential, revealed by 3D spheroid‐like formation and increased growth rate in xenografts of tumors in nude mice (Figure 2). This augmentation in cellular function can be partially attributed to CRT‐mediated rewiring of network of cell‐to‐cell interaction and communication, metabolic homeostasis, and protein stability (Ca^2+^, glucose uptake, and grp78). These observations are in line with results of recent studies showing reduced proliferation and viability of melanoma, breast, kidney, colon, ovarian, and neuronal tumor cells following CRT knockdown, thereby suggesting that CRT expression in tumors facilitates proliferation, malignancy, and survival.^33^ Treatment (including ionizing radiation)‐induced translocation of CRT from endoplasmic reticulum to the cell membrane has been one of the foci of many investigations related to the in vivo response of tumors as it is known to function as an “eat me” signal, triggering immunogenic cell death.^40^ Although CRT overexpression conferred resistance against radiation‐induced death in vitro (Figure 3), a higher level of treatment‐induced CRT membrane localization may trigger a robust immunogenic response compensating the attenuated direct effect of radiation on tumor cells. This strongly suggests that the influence of CRT on the cellular responses to ionizing radiation and other stresses are likely to differ among different tumor cell types, which vary in their physiological and biological behaviors. A correlation between cellular CRT levels and radiation response suggesting its role in radioresistance demonstrated here adds to the hitherto known roles of CRT in the cellular and systemic response to radiation. However, further studies reinforcing this relationship and unraveling the underlying mechanisms are essential before strongly implicating its role in the cellular responses to IR and other therapeutic agents.

It appears that overexpression of CRT (a molecular chaperone) positively cooperates with another molecular chaperone grp78 post irradiation to elicit a robust UPR response post irradiation in CROE cells, providing a survival advantage to the cells. CRT provides dual benefit to cancer cells by upregulating prosurvival signaling (grp78) on one hand and downregulating tumor‐suppressor proteins (P53) on the other, thus playing a pivotal role in IR‐induced damage response (Figure 3D). Further, absence of any significant effect on IR‐induced CRT and acetylated H4 lysine 16 levels suggests that CRT and H4 are not the direct targets of IR, although their endogenous levels were higher, and these might operate indirectly through ER stress (Figure 3D). However, further studies evaluating the influence of CRT on other important regulators of UPR response viz. phosphorylated‐PERK, ATF6, CHOP, ERO1, PDI, Calnexin, IRE1alpha, as well as phosphorylated eIF2alpha, besides phosphorylation of AKT, need to be carried out to strongly implicate UPR response as one of the important contributing factors for CRT‐mediated alterations in cellular radiation response.

CRT has been shown to acetylate proteins using both acetyl CoA and DAMC in vitro. Our earlier studies have shown that hyperacetylation in DAMC‐treated CROE cells, including that of histones of the low‐molecular‐weight proteins (<25 kD). The supra‐additive effect of DAMC in combination with radiation in CROE cells suggests the functional implication of CRT:DAMC‐mediated protein hyperacetylation in modifying vital nuclear processes like proliferation, repair, and apoptosis (Figure 4). More importantly, a higher degree of radiosensitization by DAMC in CRT‐overexpressing CROE cells demonstrated here suggests that a combination of DAMC and ionizing radiation can enhance the direct cytotoxic effect of radiation on the irradiated tumor cells as well as trigger an immune response leading for a better local tumor control. However, this needs further investigations.

Taken together, results of the present studies demonstrate that CRT overexpression confers resistance against radiation‐induced death of glioma tumor cells (by reducing mitotic death), while polyphenolic acetates like DAMC, a preferred acetyl group donor for CRT‐mediated acetylation of proteins, can enhance the radiosensitivity by enhancing apoptosis. Thus, CRT generally overexpressed in tumors can be exploited as a target for developing adjuvants (like DAMC) to radiotherapy that directly enhances radiation‐induced death of tumor cells, besides triggering immunogenic death linked to its membrane localization following irradiation.^11^ It is to be pointed out here that results of the present studies provide only a window for showing a correlation between CRT level and radiation resistance and the potential of polyphenolic acetates (like DAMC) to sensitize tumor cells with higher levels of CRT more effectively. Further studies are warranted to elucidate the mechanisms underlying these observations and in vivo evaluation of radiosensitization by DAMC, besides response of nonmalignant (normal) cells before contemplating DAMC as an adjuvant to overcome CRT‐linked radioresistance of tumors.

## AUTHOR CONTRIBUTIONS

**Amit Verma:** Conceptualization; data curation; formal analysis; investigation; methodology; software; validation; visualization; writing‐original draft; writing‐review and editing. **Aastha Arora:** Data curation; methodology. **Anant Bhatt:** Methodology; writing‐review and editing. **Mohan Arya:** Writing‐review and editing. **Virinder Parmar:** Methodology; writing‐review and editing. **Ashok Prasad:** Methodology; writing‐review and editing. **Bilikere Dwarakanath:** Conceptualization; formal analysis; funding acquisition; investigation; project administration; supervision; validation; visualization; writing‐original draft; writing‐review and editing.

## CONFLICT OF INTEREST

The authors have stated explicitly that there are no conflicts of interest in connection with this article.

## ETHICS STATEMENT

This study was carried out in strict accordance with the recommendations in the Guide for the Care and Use of Laboratory Animals in cancer research of United Kingdom Coordinating Committee on Cancer Research (UKCCCR). The protocol was approved by the Committee on the Ethics of Animal Experiments of the Institute of Nuclear Medicine and Allied Sciences (INMAS), Defence Research and Development Organization (DRDO) (Institutional Ethical committee number under which this study has been approved is INM/IAEC/2011/08/001). All efforts were made to minimize suffering of mice during sacrifice. Mice were euthanized using cervical dislocation after completion of the study.

## Data Availability

The data that support the findings of this study are available on request from the corresponding author. The data are not publicly available because of privacy or ethical restrictions.
